# Circulating Exosomal Cargoes and Platelet-Derived Factors May Contribute to Organ Failure in Sepsis

**DOI:** 10.34172/apb.47033

**Published:** 2026-05-19

**Authors:** Muhammad Esa, Sunisa Kaewpaiboon, Tawseef Ahmad, Muhammad Khan, Asad Ur Rahman, Teerapol Srichana

**Affiliations:** ^1^Drug Delivery System Excellence Center, Department of Pharmaceutical Technology, Faculty of Pharmaceutical Sciences, Prince of Songkla University, Hat Yai, Songkhla 90110, Thailand; ^2^Department of Clinical Pharmacy, Faculty of Pharmaceutical Sciences, Prince of Songkla University, Hat Yai, Songkhla 90110, Thailand; ^3^Federal Urdu University of Arts, Science, and Technology, Karachi, 75300, Pakistan; ^4^Chakri Naruebodindra Medical Institute, Faculty of Medicine Ramathibodi Hospital, Mahidol University, Samut Prakan 10540, Thailand

## To Editor,

 Sepsis is a life-threatening condition arising from a dysregulated host immune response to infection, resulting in systemic inflammation and organ dysfunction. According to the Global Burden of Disease (GBD) estimates, in 2017 there were approximately 48.9 million sepsis cases worldwide, with 11.0 million deaths, accounting for 19.7% of all global deaths.^1^ Despite advances in supportive care, effective early risk stratification and targeted therapeutic interventions remain limited.

 Emerging evidence increasingly implicates extracellular vesicles (EVs), particularly exosomes (30-150 nm), as central mediators in sepsis pathophysiology.^2,3^ The growing body of research suggests that circulating exosomes facilitate intercellular communication that influences inflammatory signaling, immunological dysregulation, endothelial damage, and multi-organ failure by carrying bioactive proteins and microRNAs (miRNAs).^4,5^ In 2019 and 2020, circulating exosomes were first identified as prognostic biomarkers, later identified as active mechanistic drivers of disease, and more recently have been employed as therapeutic delivery systems, according to studies published between 2019 and 2025, which show a clear conceptual and chronological progression(Table 1).

**Table 1:** Chronological Progression of Exosome Research in Sepsis (2019–2025)

**Year**	**Primary Focus**	**Model / Cohort**	**Exosome Source / Cargo**	**Key Findings**	**Scientific Advancement**	**Ref.**
2019	Neuroinflammation and brain injury in sepsis	CLP-induced septic rats	Adipose-derived MSC exosomes	Suppressed systemic and cerebral inflammation, apoptosis, oxidative stress, and BBB damage	First evidence of organ-protective MSC-exosomes in sepsis	^6^
2020	Disease severity and mortality	220 sepsis patients	Circulating plasma exosomes (total levels)	Exosome levels increased with sepsis severity; correlated with SOFA score; predicted 28- and 90-day mortality	Established exosomes as prognostic biomarkers	^2^
2020	Early inflammatory signaling	ED sepsis patients	Exosomal proteome (SAA-1, SAA-2, CRP)	Distinct acute-phase protein signature in septic exosomes	Demonstrated early diagnostic potential	^7^
2020	Platelet–neutrophil crosstalk	Septic shock patients + CLP mice	Platelet-derived exosomes (HMGB1, miR-15b-5p, miR-378a-3p)	Promoted NET formation via Akt/mTOR autophagy; caused lung injury	Identified exosomes as active drivers of tissue damage	^4^
2023	Prognostic miRNA profiling	Sepsis patients + validation cohort	Exosomal miRNAs (let-7f-5p, miR-331-3p, miR-301a-3p, miR-335-5p)	Strong prediction of in-hospital and 90-day mortality	Molecular prognostication	^5^
2023	Endothelial dysfunction and ARDS	Sepsis patients	EV caspase-1, miR-126	EV-induced EC injury linked to ARDS, ARF, and mortality	Linked exosomes to vascular failure	^8^
2024	Immune cell survival and AKI	LPS-treated T cells + SAKI mice	HUCMSC-exosomes delivering miR-375	Promoted autophagy, reduced T-cell apoptosis via HDAC4 suppression	Showed immune-restorative exosome therapy	^9^
2025	Diagnostic discrimination of shock	Postsurgical ICU patients	EV-miRNAs (miR-100-5p, miR-148a-3p, miR-451a)	Distinguished septic from non-septic shock with high accuracy	Enabled early septic shock diagnosis	^10^
2025	Lung–systemic communication	Sepsis ± ARDS patients	EV-miRNAs (miR-766, miR-127, miR-29b, miR-885-5p, etc.)	Identified ARDS-specific miRNA signatures targeting GP6 signaling	Clarified lung-origin EV signaling	^11^
2025	Hepatic and renal injury mechanisms	Septic rats + patients	Serum exosomal miR-122-5p	Activated TAK1/SIRT1/NF-κB, causing liver and kidney injury	Defined pathogenic exosomal miRNA axis	^3^
2025	Metabolic reprogramming therapy	CLP-induced ALI mice	FGF21-loaded M2 macrophage exosomes	Reduced inflammation, glycolysis, apoptosis; promoted M2 polarization	Advanced precision metabolic exosome therapy	^12^

**Abbreviations:** AKI, acute kidney injury; ALI, acute lung injury; ARDS, acute respiratory distress syndrome; ARF, acute renal failure; Akt, protein kinase B; BBB, blood–brain barrier; CLP, cecal ligation and puncture; CRP, C-reactive protein; EC, endothelial cell; ED, emergency department; EVs, extracellular vesicles; FGF21, fibroblast growth factor 21; GP6, glycoprotein VI; HDAC4, histone deacetylase 4; HMGB1, high-mobility group box 1; HUCMSC, human umbilical cord-derived mesenchymal stem cell; ICU, intensive care unit; LPS, lipopolysaccharide; MAPK, mitogen-activated protein kinase; miRNA/miR, microRNA; mTOR, mammalian target of rapamycin; MSC, mesenchymal stem cell; NETs, neutrophil extracellular traps; NF-κB, nuclear factor kappa B; PI3K, phosphoinositide 3-kinase; SAKI, sepsis-associated acute kidney injury; SAA-1/SAA-2, serum amyloid A-1/A-2; SOFA, Sequential Organ Failure Assessment; srIκBα, super-repressor inhibitor of NF-κB alpha; TAK1, transforming growth factor-β-activated kinase 1.

 In a septic rat model, Changet al. (2019) first demonstrated that adipose-derived mesenchymal stem cell–derived exosomes conferred significant neuroprotection. Administration of exosomes significantly (*P* < 0.0001) reduced circulating inflammatory cells and pro-inflammatory cytokines (IL-6 and TNF-α) at 6–72 h after sepsis induction. It also downregulated inflammatory (NF-κB, TLR2/4, HMGB1), apoptotic (cleaved caspase-3, PARP, Bax), and oxidative stress markers (NOX-1/NOX-2), while increasing regulatory T-cell populations and attenuating brain injury biomarkers (AQP4 and γ-H2AX).^6^ Clinical studies also started to demonstrate the diagnostic and prognostic value of circulating exosomes during this period. The first extensive clinical data connecting circulating plasma exosomes to sepsis severity and outcome were presented by Imet al. in 2020. Overall plasma exosome levels in a group of 220 septic patients showed a positive correlation with Sequential Organ Failure Assessment (SOFA) scores and gradually increased from controls to sepsis and septic shock. Increased exosome levels were found to be an independent predictor of 28- and 90-day mortality, and the study concluded that circulating exosomes may serve as early markers of organ failure and death.^2^ Morriset al. (2020) study showed that exosomes from septic emergency department patients (n = 7) have unique proteomic signatures, extending these findings to the early phases of illness. Proteomic analysis of 261 proteins revealed that 62 proteins (23.8%) were differentially expressed between septic patients and healthy controls (*P* < 0.05), of which 23 proteins remained significant after false discovery rate adjustment (*P* < 0.05). Serum amyloid A-1, serum amyloid A-2, and C-reactive protein were among the acute-phase and inflammatory proteins that were significantly enriched in proteomic profiling, suggesting that exosomes represent early systemic inflammatory responses even at the initial clinical presentation.^7^

 Later, attention subsequently shifted toward exosomal cargo specificity, particularly miRNAs and their utilization as prognostic and diagnostic biomarkers. Shinet al. (2023) identified a distinct pattern of downregulated plasma exosomal miRNAs in sepsis patients (n = 35), including hsa-let-7f-5p, miR-331-3p, miR-301a-3p, and miR-335-5p, all of which were significantly reduced compared with healthy controls (*P* < 0.0001). The pathogenesis of sepsis was linked by a bioinformatics study to the PI3K-Akt and MAPK signaling pathways, and these miRNAs showed excellent prediction performance for in-hospital and 90-day mortality.^5^ Liet al. (2023) investigated septic patients (n = 96) and found that circulating EVs with elevated caspase-1 activity and declining levels of miR-126 were significantly associated with endothelial dysfunction, acute respiratory distress syndrome, acute renal failure, and mortality. The study identified an important relationship between the dynamic nature of EV-miRNA changes over time and sepsis-related organ failure ^8^. A three-miRNA EV-derived signature (miR-100-5p, miR-148a-3p, and miR-451a) was found to differentiate septic shock from non-septic shock with superior diagnostic accuracy in a prospective examination of postoperative shock patients (n = 52).^10^

 The molecular role of exosomes as organ injury drivers has been further clarified by multiple studies. In one study, platelet-derived exosomes were found to trigger lung damage and worse outcomes during septic shock by promoting excessive neutrophil extracellular trap (NET) production via exosomal HMGB1 and miR-15b-5p/miR-378a-3p. *In vivo*, exosome release, NET formation, and lung damage were all reduced by platelet depletion.^4^ In a cohort of septic patients with (n = 5) and without acute respiratory distress syndrome (n = 13), EV-miRNA signatures (miR-766, miR-127, miR-29b, miR-885-5p) were associated with sepsis-induced respiratory distress syndrome.^11^ A more recent study by Wanget al. (2025), found that serum exosomal miR-122-5p induces hepatic and renal injury via activation of the TAK1/SIRT1/NF-κB pathway, further verifying organ-specific injury mediated by exosomal miRNAs. In addition, they found that exosome release or miR-122-5p inhibition significantly lowers organ damage and inflammation.^3^

 Unlike pathogenic role, two different animal studies reported that exosomes derived from mesenchymal stem cells^9^, or macrophages^12^ showed protective effects in sepsis-related organ injury. Liu & Chen (2024) found that miR-375 can be delivered by human umbilical cord-derived mesenchymal stem cell exosome to decrease histone deacetylase-4 (HDAC4), promoting autophagy, and prevent CD4⁺ T-cell apoptosis, thereby protecting the kidneys from acute renal damage caused by sepsis in C57BL/6 mice.^9^ A different study by Wu et al. (2025) found that fibroblast growth factor 21 (FGF21)-loaded M2 macrophage-derived exosomes alleviated sepsis-induced acute lung injury by promoting M2 polarization, inhibiting glycolysis, and reducing apoptosis.^12^ Notably, only one study employed engineered exosomes as an active therapeutic delivery platform optogenetically loaded super-repressor IκB (srIκB) in sepsis therapy. Treatment with srIκB-loaded exosomes markedly improved survival in both lipopolysaccharide and cecal ligation and puncture-induced sepsis models, reduced circulating pro-inflammatory cytokines (TNF-α, IL-1β, IL-6, and chemokine CCL4), and decreased sepsis-associated acute kidney injury, with histological injury scores reduced by approximately 50%.^13^

## Conclusion

 Published evidence from 2019 to 2025 delineates a significant paradigm shift in our understanding of EVs in sepsis. Circulating exosomes were initially recognized as prognostic biomarkers, subsequently characterized as active mediators of disease pathophysiology and, more recently, have been explored as therapeutic delivery systems. Circulating exosomes carrying cargoes such as miR-127, miR-15b-5p, miR-29b, miR-378a-3p, miR-766, and miR-885-5p, along with platelet-derived HMGB1, and inflammatory proteomic signatures may collectively be associated with sepsis-induced organ injury. As the field matures, the integration of exosomal profiling and cargo-specific analysis into intensive care workflows may enable earlier diagnosis and improved mortality stratification in sepsis. Although the International Society for Extracellular Vesicles (ISEV),^14^ established in 2011-2012, aims to advance EV research globally and promote standardization and innovation, further efforts are required. Therefore, we believe that in order to ensure clinical translatability, future research must place more attention to the systematic development of exosome-based drug delivery strategies, and the standardized quantitative profiling of exosomal cargo from patients with sepsis.

## Competing Interests

 The authors declare that they have no conflicts of interest. No funding or financial support was received from any organization, industry, or external source for the conduct of this work

## Data Availability Statement

 Data sharing is not applicable to this work, as no datasets were generated or analyzed during the preparation of this manuscript.

## Ethical Approval

 Not applicable. As this work is presented as a “Letter to the Editor”, ethical approval was neither sought nor required.
